# Pseudoaneurysm following hamstring tendon harvest in arthroscopic anterior cruciate ligament reconstruction: a case report

**DOI:** 10.1186/s12891-020-03721-4

**Published:** 2020-10-21

**Authors:** Chung-Wei Ho, Shih-Han Lee, Shen-Han Wu, Chun-Yu Lin, Chian-Her Lee, Jia-Lin Wu

**Affiliations:** 1 Department of Orthopedics, School of Medicine, College of Medicine, Taipei Medical University, Taipei, Taiwan; 2 Department of Orthopedics, Taipei Medical University Hospital, Taipei, Taiwan; 3 Department of Medical Imaging, School of Medicine, College of Medicine, Taipei Medical University, Taipei, Taiwan; 4 Department of Medical Imaging, Taipei Medical University Hospital, Taipei, Taiwan

**Keywords:** ACL reconstruction, Case report, Complication, Hamstring tendon harvest, Pseudoaneurysm

## Abstract

**Background:**

Vascular injury is a very rare complication following arthroscopic knee surgery. This is the first report of pseudoaneurysm at the saphenous branch of the descending genicular artery complicating semitendinosus tendon harvest in arthroscopic anterior cruciate ligament reconstruction.

**Case presentation:**

A 19-year-old male had developed large ecchymosis, focal swelling and tenderness around his posteromedial knee after an arthroscopic anterior cruciate ligament reconstruction. Compartment syndrome of the lower leg and deep vein thrombosis were ruled out. A pseudoaneurysm formation was confirmed through an angiography and coil embolization was performed. At one year follow up, the patient reported improved functional outcome with good stability of the knee. However, mild paresthesia over the posteromedial calf was noted due to the compression injury of the saphenous nerve by the hematoma.

**Conclusions:**

The pseudoaneurysm was presumed to result from accidental vascular injury while dissecting the accessory bands of the semitendinosus and was successfully treated by coil embolization. Care must be taken to section the expansions of the hamstring tendon, especially when the patient presents with underlying coagulopathy or vascular disease.

## Background

Isolated anterior cruciate ligament (ACL) tears are one of the most common orthopedic injuries, with an annual incidence of 68.6 per 100,000 person-years [1]. Surgery is recommended for young active patients who have a high predicted risk of recurrent instability and for individuals of all ages with demonstrated recurrent instability [2]. Arthroscopic reconstruction of the ACL alone accounts for 75,000 cases per year in the United States [3]. Vascular injuries following this surgery are especially rare and account for of < 1% of all presented complications [4]. In this paper, we report on the first case of pseudoaneurysm occurring at the saphenous branch of descending genicular artery after arthroscopic ACL reconstruction by using the semitendinosus autograft and single-tunnel technique.

## Case presentation

A 19-year old male had a twisting injury to his left knee with a popping sensation when landing during a basketball game. The patient was otherwise healthy, but his history was notable for epistaxis lasting more than 30 min since he was 4 years old. This had been evaluated by an otolaryngologist without abnormality and there was no family history of bleeding or clotting disorder. A coagulation test revealed normal prothrombin time (13.4 s; reference interval: 11.0–14.5 s), international normalized ratio (1.05) and activated partial thromboplastin time (40.7 s; reference interval: 32.0–45.1 s). Physical examination including Lachman test, anterior drawer, and pivot shift indicated an acute ACL injury and an MRI confirmed the diagnosis without concomitant injuries. An arthroscopically assisted ACL reconstruction with hamstring tendon autograft was delayed approximately 1 month to allow swelling to subside and to regain range of motion.

A tourniquet was inflated to 280 mmHg prior to the surgery. A longitudinal 3-cm incision for the harvest was made 2 cm medial to the medial edge of the tibial tubercle, centered over the palpable pes tendons. The distal end of the semitendinosus was whipstitched with no.2 sutures (Ethibond, Ethicon, Somerville, NJ, USA) and detached from tibia. When releasing expansions of the semitendinosus tendon, the scissors were remained opened and pushed proximally to avoid accidental cutting of the tendon tissue. We performed circumferential palpation around tendons to confirm full release. A closed end tendon stripper is then passed with slow steady force in line with the tendon’s trajectory with the knee flexed. The graft is carefully moved from the harvest site to the back table for graft preparation. A two-portal arthroscopic technique was performed with independent tibial and femoral tunnel drilling. The 4-strand hamstring graft was secured with an endobutton on the femur and Bioscrew on the tibia. Hemostasis was achieved throughout the procedure. After incision closure, the tourniquet was deflated without abnormal bleeding being noted and the tourniquet time was 105 min. He was then discharged uneventfully.

**Fig. 1:**
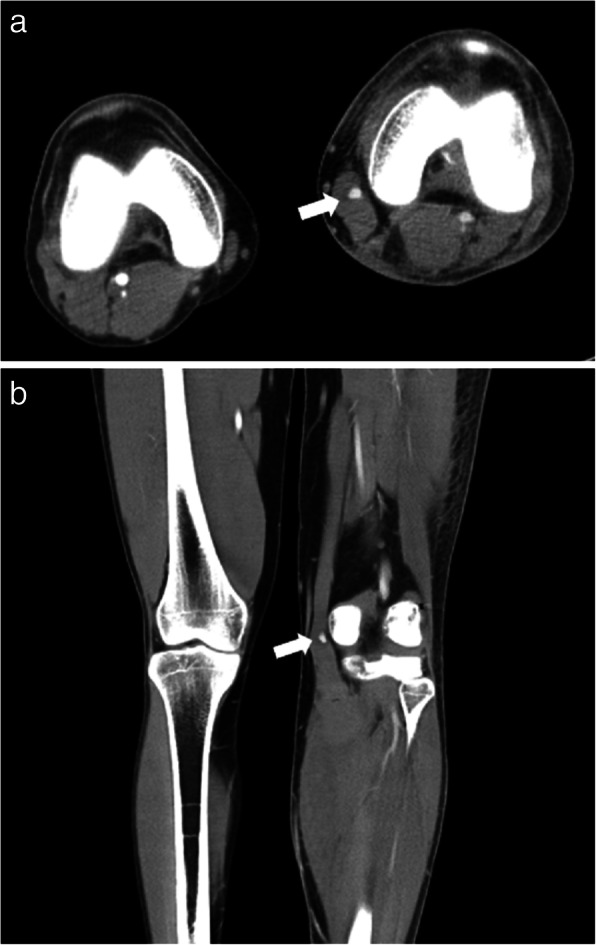
Computed tomography angiography of the lower limbs. **a** Axial view and **b** coronal view showed intramuscular hematoma and unusual focal contrast enhancement (white arrows) within the sartorius

Large ecchymosis around his posteromedial knee with focal swelling and tenderness was observed on the sixth postoperative day at the outpatient department. He reported no throbbing pain or pulsatile mass. Physical examination revealed intact distal pulses and there was no pain on passive stretching of his ankle. Deep vein thrombosis was ruled out by a negative D-dimer test and Doppler ultrasound image. Bleeding or a pseudoaneurysm formation within the sartorius was impressed on the computed tomography image (Fig. 1).

**Fig. 2:**
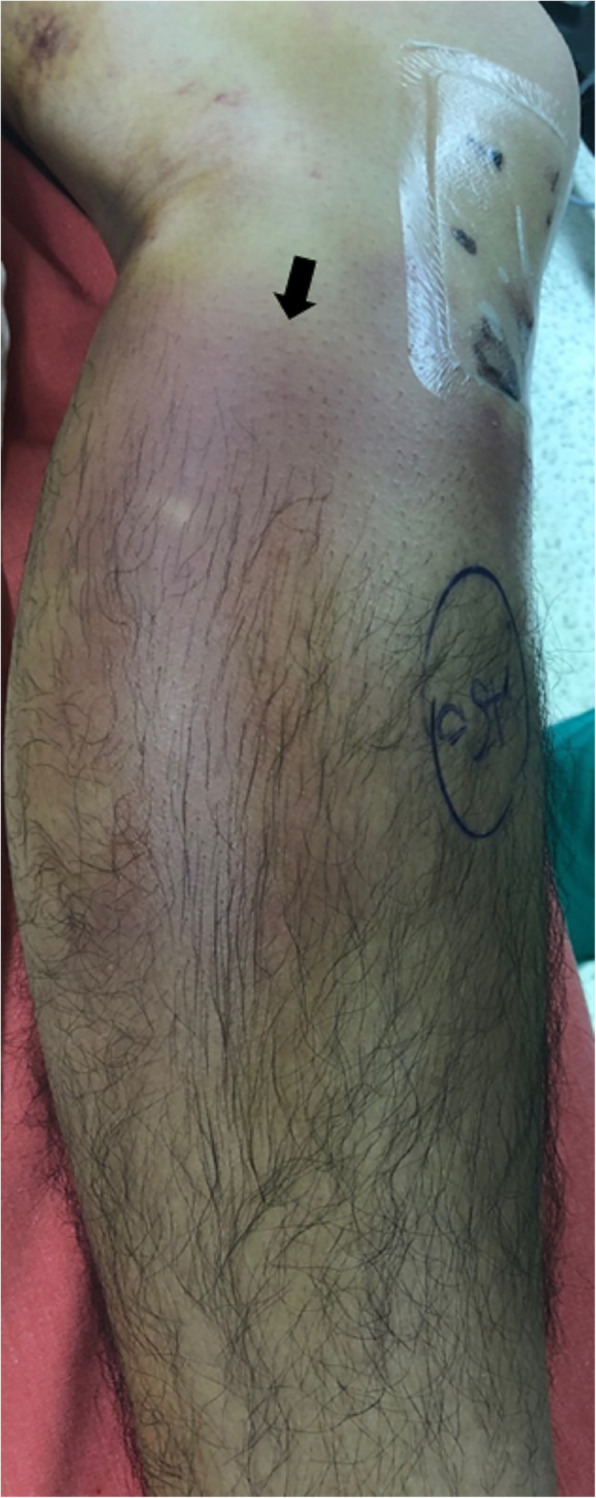
Appearance of the left knee upon readmission. Large ecchymosis (black arrow) with focal swelling and tenderness persisted at the posteromedial calf postoperatively

**Fig. 3:**
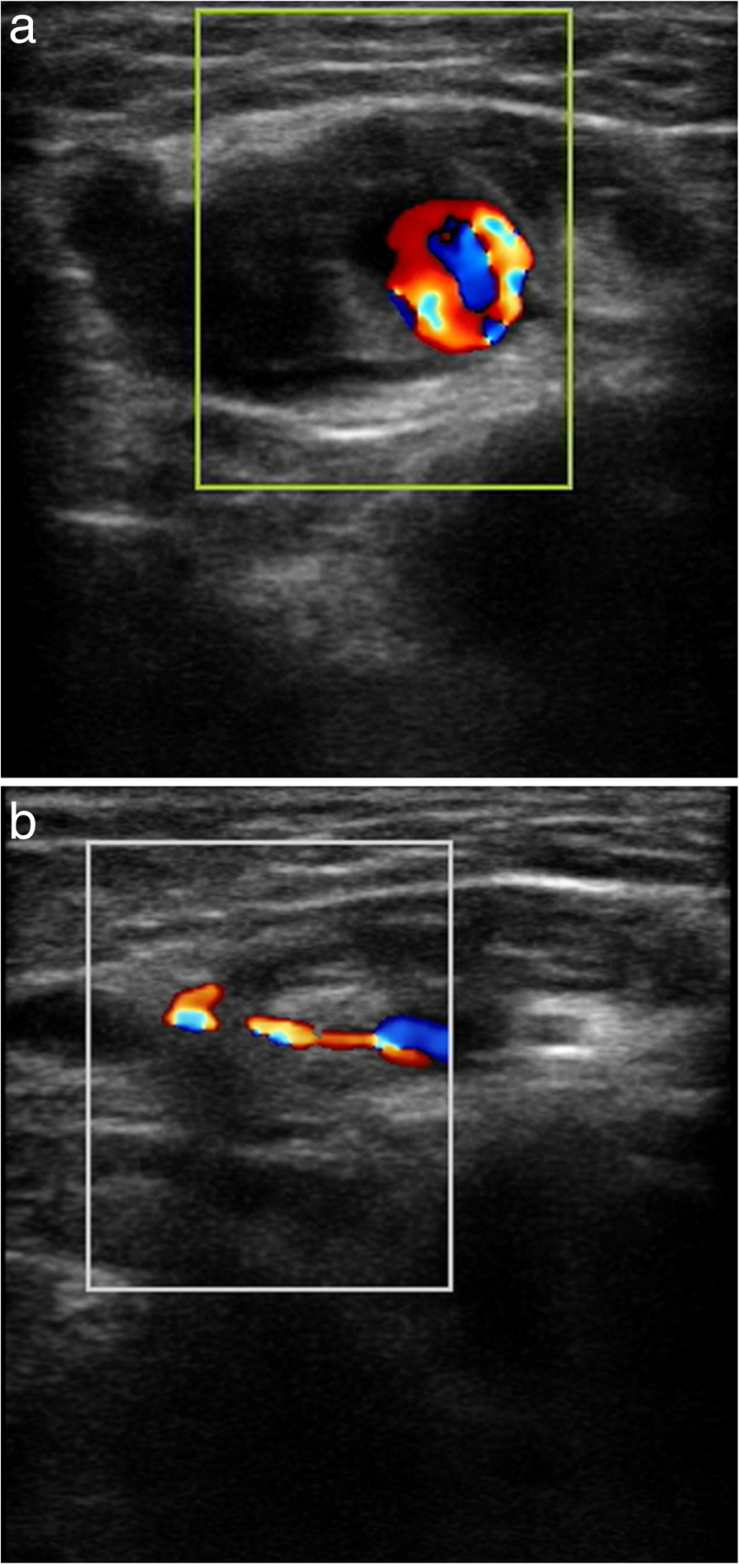
Doppler ultrasonography over the posteromedial aspect of the left knee. **a** A well-defined mass with bidirectional flow was shown. **b** The size of the mass decreased under compression with the ultrasound probe

**Fig. 4:**
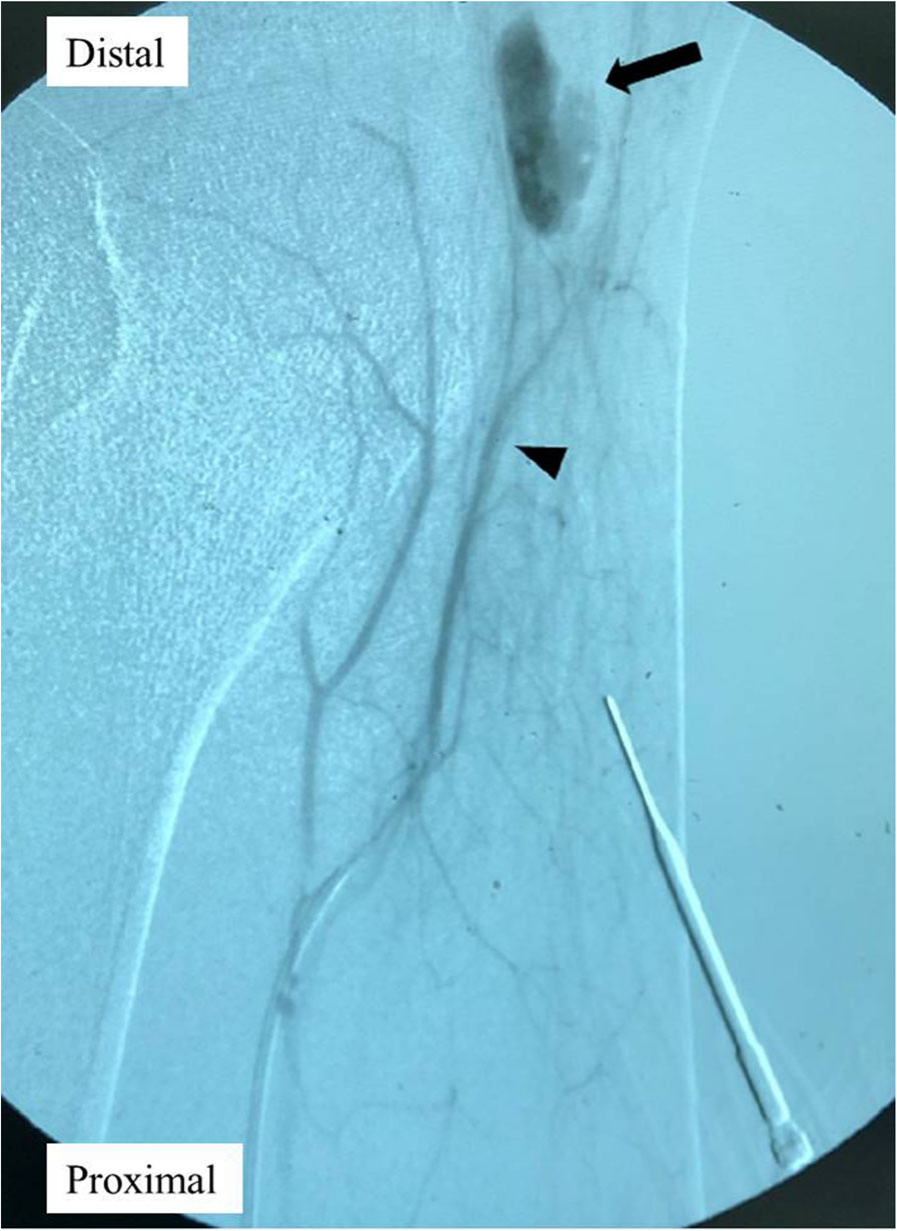
Diagnostic angiography of the left knee. A pseudoaneurysm with active extravasation (black arrow) was noted adjacent to the saphenous branch of the descending genicular artery (black arrowhead)

**Fig. 5:**
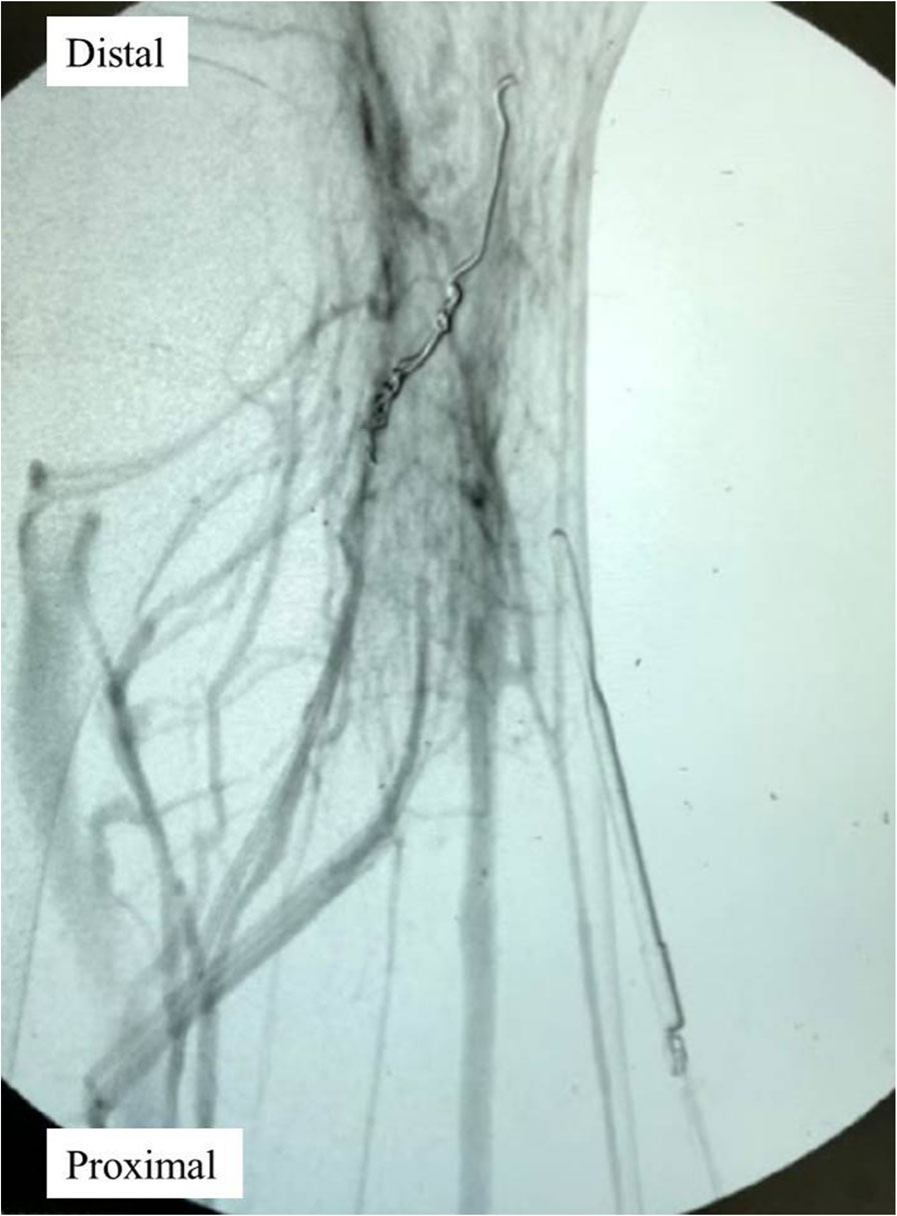
Post-embolization angiography of the left knee. The pseudoaneurysm was devascularized after placement of two micro-coils (TORNADO 3–2 mm, Cool Medical)

Due to the persistent symptoms, he was readmitted to our ward for further management (Fig. 2). A repeated Doppler ultrasound showed a compressible pseudoaneurysm over the posteromedial aspect of the knee (Fig. 3). It was confirmed at the saphenous branch of the descending genicular artery in angiography (Fig. 4). Coil embolization was chosen over open ligation or excision due to the terminality of this arteriole. Devascularization of the pseudoaneurysm was achieved by placement of two micro-coils (TORNADO 3–2 mm; Cool Medical; Fig. 5). Open debridement with drainage through a new 1-cm incision centered over the ecchymosis was then performed and 600 mL of hematoma was removed.

No sign of arterial insufficiency or edema was observed after the procedure. At 1 year follow up, the International Knee Documentation Committee Subjective Knee Form score had improved from 6.9 preoperatively to 66.7, and the stability recovered well with negative Lachman and pivot shift tests. He has continued to play basketball recreationally. Mild paresthesia over the posteromedial calf was noted due to the compression injury of the saphenous nerve by the hematoma.

## Discussion and conclusions

According to the few reports on pseudoaneurysm following arthroscopic ACL reconstruction with hamstring tendon autograft, popliteal artery is the most frequently injured vessel [5–8]. Sporadic vascular injuries were also found at different small branches including the articular branch of the descending genicular artery, the perforating branch of the deep femoral artery, and the medial inferior genicular artery [9–13]. Vincent et al. described a pseudoaneurysm at the saphenous branch of the descending genicular artery after arthroscopic meniscectomy, which was caused by accessory medial portal construction and treated with ligation [14]. To the best of our knowledge, no report has been published regarding pseudoaneurysm at the saphenous branch of the descending genicular artery following arthroscopic ACL reconstruction.

**Fig. 6:**
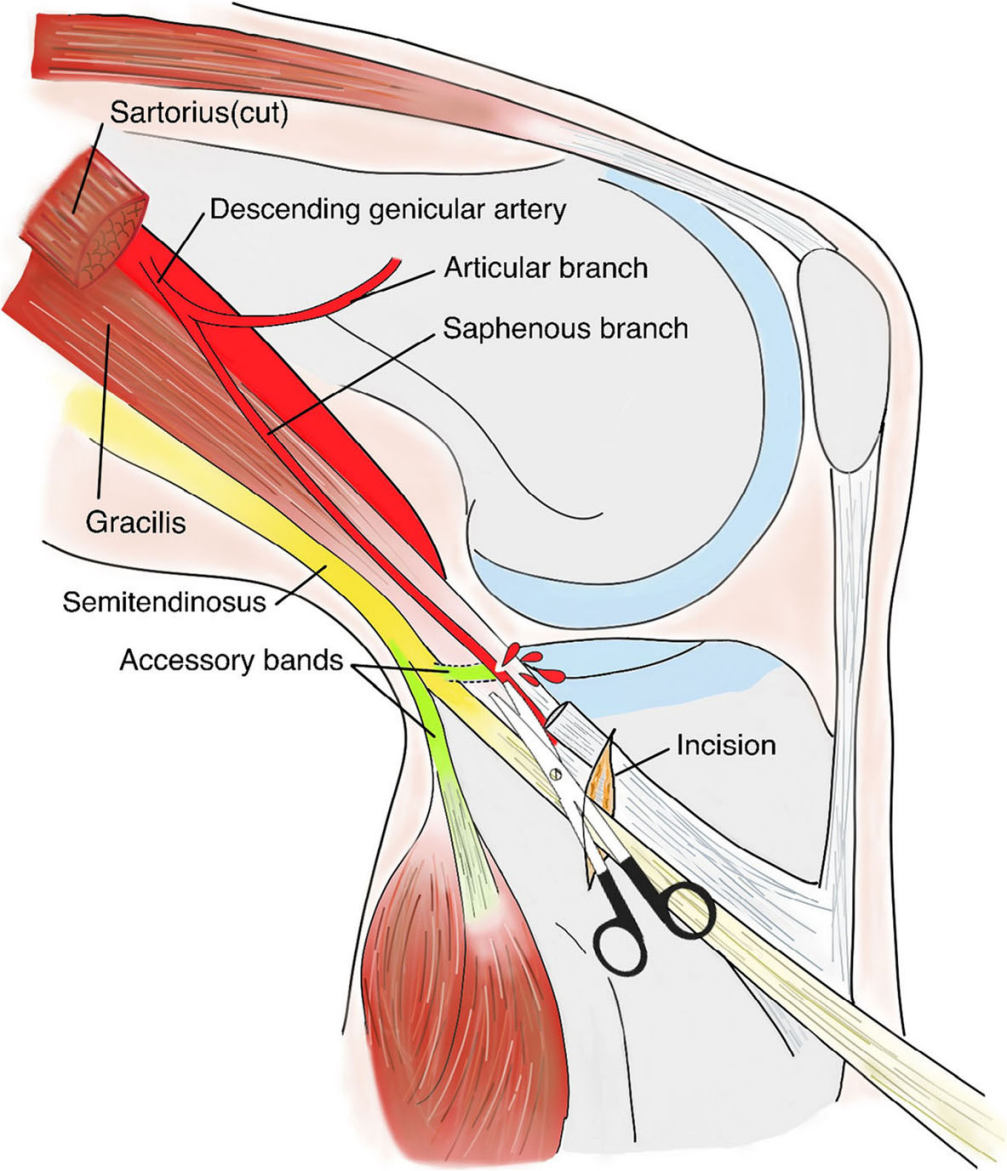
Illustration of the mechanism of vascular injury. The saphenous branch of the descending genicular artery was damaged when the expansions of semitendinosus were sectioned with opened scissors

We believed that the saphenous branch of the descending genicular artery was traumatized during tendon harvest. Several thick fascial bands pass between semitendinosus and gracilis and also from these hamstring tendons to gastrocnemius, popliteal, pretibial, and superficial fascia [15]. Identifying and dividing these structures to avoid premature tendon amputation and short graft are crucial. The saphenous branch of the descending genicular artery generally passes in a posteromedial location between sartorius and gracilis and crosses the medial joint line in an approximately vertical direction [16]. Its close anatomical proximity to the targeted tendon autograft produces certain risk of vascular injury while dissecting the expansions (Fig. 6).

Advancing the opened scissors proximally in a blind fashion is arguably the most dangerous maneuver in this technique. Also, pushing a closed end tendon stripper with an inappropriate direction not following the hamstring trajectory may cause unwanted shear force on the surrounding vessels. Minor injury to arterioles could be easily neglected during the procedure because vessel retraction under inflated tourniquet results in minimal bleeding [17]. In addition to surgical injury, the underlying disease of patients such as atherosclerotic peripheral vascular disease and bleeding diathesis makes them susceptible to pseudoaneurysm formation [4]. Regarding the history of frequent and copious epistaxis in our case, it is suggestive of a bleeding tendency and a higher chance of developing vascular complication in spite of his normal coagulation studies.

The reported vascular complication does not only concern ACL reconstruction but generally the hamstring tendon harvest in any surgical procedure. It is preferred to reveal and divide the fascial bands one after the other under visual control by pulling on the targeted tendons [18]. Endoscopic harvest technique has been described by Yeh et al. demonstrating a clearer assessment of more proximal fascial and accessory bands and neurovascular structures [19]. More careful hemostasis throughout the surgery is warranted. Deflating the tourniquet before wound closure may help alert the possibility of vascular injury. Intra-arterial interventions enable precise treatment to a more complex and difficult pseudoaneurysm at deep feeding vessels [4]. The sufficient anastomosis around the knee allows embolization without risk of skin or tissue necrosis.

In conclusion, although vascular injury rate remains very low in arthroscopic knee surgeries, the possibility of pseudoaneurysm formation at the saphenous branch of descending genicular artery during tendon harvest should not be overlooked. Care must be taken to section the expansions of the hamstring tendon, especially when the patient presents with underlying coagulopathy or vascular disease. Coil embolization has been an effective treatment option to the pseudoaneurysm at terminal branches.

## Data Availability

Data sharing is not applicable to this article as no datasets were generated or analyzed during the current study.

## Abbreviation

ACL: Anterior cruciate ligament
